# Salvage surgery following primary treatment in recurrent vestibular schwannoma: surgical outcomes and progression-free survival- a meta-analysis

**DOI:** 10.1007/s11060-026-05629-w

**Published:** 2026-05-29

**Authors:** Lisa Haddad, Felix Arlt, Erdem Güresir, Johannes Wach

**Affiliations:** 1 Comprehensive Cancer Center Central Germany, Partner Site Leipzig, 04103 Leipzig, Germany; 2 Department of Neurosurgery, Leipzig University, University Hospital Leipzig, Liebigstraße 20, 04103 Leipzig, Germany

**Keywords:** Recurrence, Vestibular schwannoma, Postoperative outcome

## Abstract

**Background:**

Vestibular schwannomas (VS) are benign tumors of the eighth cranial nerve. While gross total resection (GTR) is often achieved in primary surgery, recurrence remains a therapeutic challenge. Repeat microsurgery is a key treatment option, but evidence is limited and inconsistently reported. This study systematically synthesizes current data on outcomes after repeat microsurgical resection for recurrent VS.

**Methods:**

A systematic PubMed search identified studies reporting outcomes of repeat microsurgery for recurrent VS. Extracted data included patient characteristics, GTR rate, progression-free survival (PFS), facial nerve function (House–Brackmann scale), hearing preservation, postoperative complications, and hydrocephalus or shunt dependency. Pooled analyses using random-effects models estimated summary proportions across studies.

**Results:**

Ten studies including 359 patients met inclusion criteria. The pooled GTR rate was 0.71 (95% CI: 0.46–0.87). Four studies (49 patients) reported PFS, with no tumor recurrences (pooled proportion = 1.00). Postoperative facial nerve deterioration occurred in 43% (95% CI: 0.29–0.58). Cerebrospinal fluid (CSF) leakage was the most frequent complication (11% (95% CI: 0.05–0.21)). Between-study heterogeneity was low to moderate in most of the studies (I² = 0–38.6%).

**Conclusions:**

Repeat microsurgery for recurrent VS provides good tumor control but carries higher risks of facial nerve deterioration and other complications compared with primary surgery. Current evidence is limited by small cohorts and retrospective designs. Standardized reporting and prospective multicenter studies are required to improve treatment strategies for this rare and challenging condition.

**Supplementary Information:**

The online version contains supplementary material available at 10.1007/s11060-026-05629-w.

## Introduction

Vestibular schwannomas (VS), benign tumors of the vestibular branch of the eighth cranial nerve may be managed by observation, microsurgical resection, fractionated radiotherapy, and stereotactic radiosurgery (SRS); depending on tumor size, symptoms and institutional expertise [1].

Although gross total resection (GTR) is typically the goal, the risk of true tumor recurrence after complete resection remains low, with reported rates around 3–4% [2]. In contrast, recurrence or progression is substantially more common after less complete resections, occurring in approximately 9.5% after near-total resection (NTR; 6 of 63 cases) and up to 30.8% following subtotal resection (STR) [2]. These differences underscore the importance of long-term surveillance, particularly in cases without GTR.

Salvage surgery following primary treatment, when required, is technically demanding due to scarring and adhesions—especially around the facial nerve—and is therefore associated with increased morbidity, most notably an increased risk of facial nerve deterioration [3].

SRS achieves high tumor-control rates in recurrent or progressive cases [4], but repeat microsurgery remains essential for selected patients with large symptomatic recurrences, brainstem compression, or failed radiosurgery. Evidence is limited to retrospective single-center series [1].

Recent reports describe indications, perioperative risks, and outcomes after surgery, but heterogeneous methodologies and follow-up durations limit generalizability. This includes contemporary case series focused on salvage surgery following primary treatment cohorts with detailed facial nerve and tumor-control endpoints.

The meta-analysis aims to synthesize current evidence on outcomes after repeating microsurgical resection for recurrent VS. The primary endpoint is progression-free survival (PFS). Secondary endpoints include GTR, facial nerve outcome, hearing preservation, postoperative complications (such as cerebrospinal fluid (CSF) leakage, ischemia, or hemorrhage), hydrocephalus and shunt dependency.

## Methods

### Search Strategy and Screening

This meta-analysis was conducted in accordance with the PRISMA 2020 guidelines [5] and was prospectively registered in PROSPERO (CRD420251074685). The completed PRISMA 2020 checklist is provided in the supplementary Table S1.

A systematic literature search was performed on August 2, 2025, using the PubMed database with the following search terms in various combinations: “acoustic AND neuroma AND residual,” “vestibular AND schwannoma AND residual,” “acoustic AND neuroma AND recurrence,” “vestibular AND schwannoma AND recurrence,” “acoustic AND neuroma AND regrowth,” and “vestibular AND schwannoma AND regrowth.” There were no restrictions on publication year. Only studies published in English were included and studies involving Humans.

### Inclusion criteria

Selection followed the PICOS (population, intervention, comparator, outcomes, and study design) framework [6]: patients undergoing re-do surgical treatment. Comparators were not required, where studies compare subgroups, subgroup analysis will be conducted; all results of the prespecified endpoints were reported.

Studies were included if they: (1) reported human revision (second or further) microsurgical procedures for recurrent VS and (2) contained extractable data.

Excluded were non-English, preclinical, review, or radiotherapy-only studies, as well as those with NF2-related schwannomatosis patients, conference abstracts, or insufficient outcome data. Two independent reviewers screened all titles and abstracts for eligibility; disagreements were resolved by a third author. After the removal of 691 duplicate entries, 808 remained; 724 were excluded as irrelevant, including non-surgical management, studies on NF2-related schwannomatosis, animal models, or non-original data. 84 full-text articles were reviewed, and 72 were excluded for reasons including unavailable full text, insufficient data, lack of specification regarding recurrent VS, or exclusive focus on radiotherapy. Ultimately, 10 studies met all inclusion criteria.

### Data extraction and quality assessment

A standardized data extraction form in Microsoft Excel was used by two independent reviewers to systematically collect the relevant data. Extracted variables included study characteristics (authors, year, country, design), patient demographics (age, sex, sample size), extent of prior resection, surgical details (approach, extent of resection), follow-up duration, and all relevant clinical outcomes. The primary outcome was PFS, reported either in months or as progression-free rates at predefined follow-up intervals. Secondary outcomes were GTR, postoperative facial nerve function, measured by House-Brackmann (HB) scale [7], hearing preservation and postoperative complications (intracranial haemorrhage, postoperative ischemia, postoperative dysphagia, postoperative CSF leakage, hydrocephalus and shunt dependency). Facial nerve deterioration was defined as postoperative deterioration measured on the HB scale [7] by one point. Definitions of hearing loss and assessment time points varied; no study used objective measures like the Gardner–Robertson scale. The risk of bias was assessed independently by two reviewers using the Newcastle-Ottawa Scale (NOS) for non-randomized studies [8]. Discrepancies were resolved through discussion. The NOS ratings were used to describe study quality rather than to exclude studies, as all available data were retained to ensure a comprehensive synthesis in this rare clinical context. Full scores are reported in supplementary Table S2.

### Statistical analysis

All statistical analyses were conducted using R Studio (version 2024.12.1 + 563). PFS was summarized descriptively due to no observed events regarding second recurrence of VS, while odds ratios (ORs) for binary outcomes were calculated. A random-effects model (Der Simonian and Laird) was applied to account for potential heterogeneity among studies, quantified by I² and Chi² (with *p* < 0.10 considered indicative of significant heterogeneity) [9]. As a rule of thumb, I² values ≤ 40% are often considered unimportant, 30–60% moderate, 50–90% substantial, and 75–100% as indicative of considerable heterogeneity [9]. Forest plots were generated to visualize individual and pooled effect estimates.

## Results

### Literature search

**Fig. 1:**
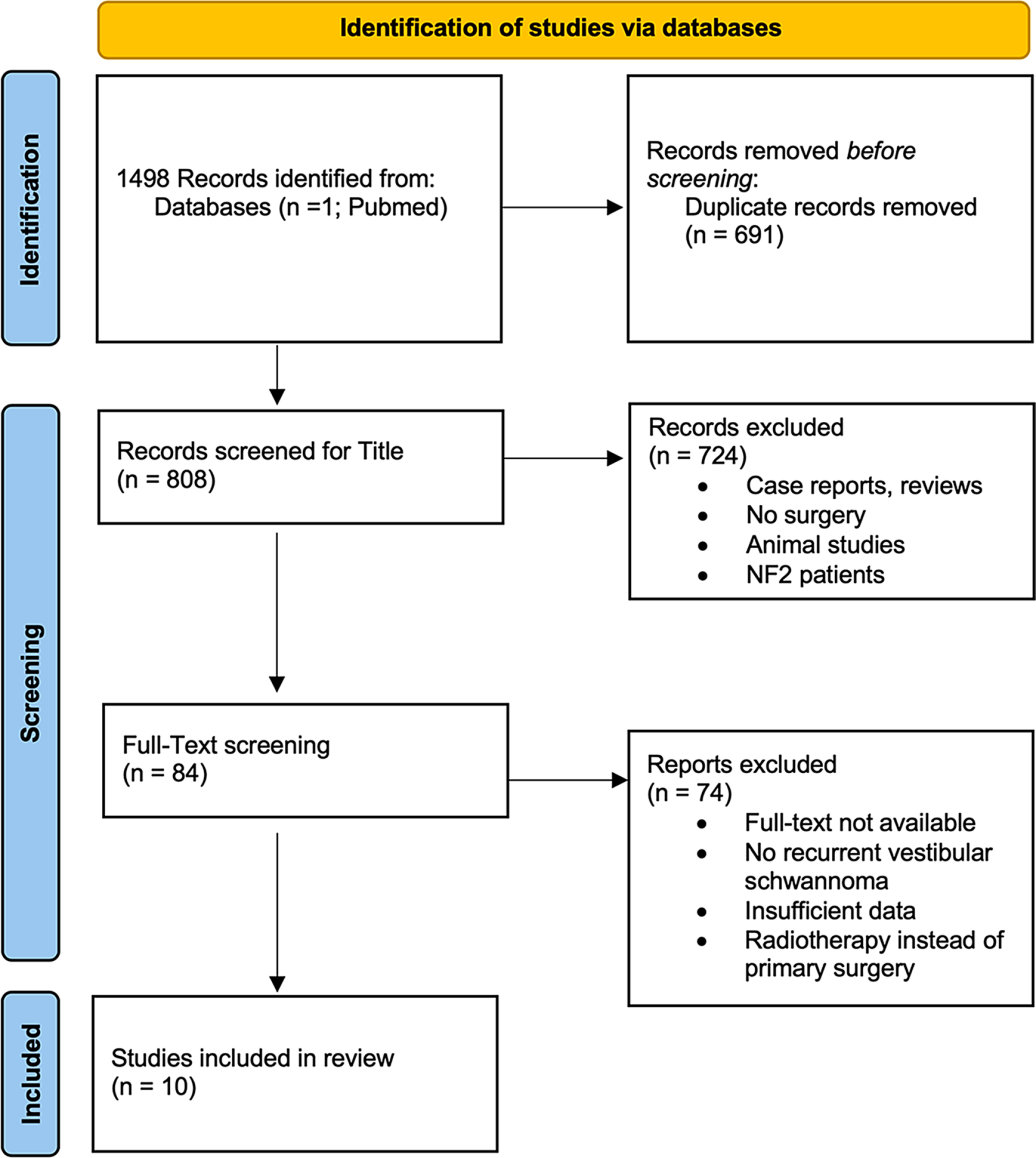
PRISMA flow diagram summarizing identification process of included studies

The systematic search yielded 1498 records, 691 duplicates were removed. After screening of the remaining 808 titles and 84 full texts 10 studies that met all inclusion criteria were included. The study selection process is summarized in the PRISMA flow diagram (Fig. 1).

### Study characteristics

The included studies were published between 1987 and 2025. All studies employed a retrospective, single-center design and focused on patients who underwent revision surgery for recurrent or residual vestibular schwannoma. An overview of study characteristics is provided in Table 1.

A total of ten studies comprising 368 patients were included. Data on surgical approaches for both primary and revision procedures were available for most included studies (Table 1). In primary surgery, the retrosigmoid (RS) approach was most frequently used, accounting for the majority of cases in several cohorts (e.g., Beatty et al. [10]: 22 RS; Freeman et al. [11]: 22 RS; Samii et al. [12]: 36 RS). The translabyrinthine (TL) and middle fossa [13] approaches were applied less frequently. In revision surgery, both RS and TL approaches were commonly employed. While RS remained predominant in some series (e.g., Samii et al. [12]: 36 RS; Hong et al. [14]: 14 RS vs. 1 TL), other studies demonstrated a higher proportion of TL approaches (e.g., Freeman et al. [11]: 19 TL vs. 8 RS; Roche et al. (Roche, 2008 #19826): 9 TL vs. 1 RS; Peng et al. [15]: 217 TL vs. 6 RS and 13 MF). MF and transcochlear (TC) approaches were used in selected cases only. Some studies demonstrated consistency in approach selection (e.g., RS-to-RS), while others suggested a shift from RS in primary surgery toward TL in revision cases. However, a patient-level comparison of approach sequences was not consistently reported and could not be systematically assessed.

Previous treatments consisted of subtotal or gross total microsurgical resections, with three studies also including patients previously treated with radiotherapy (Kunert et al. [16], Nandoliya et al. [17], Peng et al. [15], Roche et al. (Roche, 2008 #19826)). For detailed information see Table 1.

Follow-up durations varied across studies, ranging from a Median or Mean of 30 days to 116 months when reported. Definitions of recurrence and reported outcomes differed slightly, with most studies evaluating facial nerve function, progression-free survival, and common postoperative complications such as CSF leak, hydrocephalus, hearing impairment, dysphagia, or bleeding. Shunt dependency was reported in two studies only (Freeman et al. [11], Peng et al. [15]). For detailed information see Table 2.

No prospective or randomized designs were identified among the included literature.

**Table 1:** Characteristics of the study design, patient characteristics, prior treatments, surgical approach, follow-up periods, and main reported outcomes of the included studies

Author (Year)	Country	Study Design	*n*	Prior treatment	Surgical approach first surgery	second surgery		Follow-Up	limitations
Beatty et al. (1987)	USA	Retrospective, single-center	23	Previous surgery	22 RS; 1 ML	17 RS; 6 RS + TL		NA	3 patients with third and fourth surgery included; 8 patients with residuum, not recurrence
Freeman et al. (2021)	USA	Retrospective, single-center	27	Previous surgery	22 RS; 2 TL; 3 ML	8 RS; 19 TL		Median: 116 months	
Hong et al. (2020)	South Korea	Retrospective, single-center	15	Previous surgery	NA	14 RS; 1 TL		Mean: 80 months	
Kay Rivest et al. (2021)	USA	Retrospective, single-center	9	Previous surgery	4 RS; 4 TL; 1 ML	1 RS; 8 TL		Median: 99 months	
Kunert et al. (2014)	Poland	Retrospective, single-center	5	Previous surgery and or radiotherapy (n = NA)	5 RS	1 RS; 3 TL; 1 ML		Mean: 33 months	Not only operated patients
Nandoliya et al. (2020)	India	Retrospective, single-center	6	Previous surgery and or radiotherapy (*n* = 3)	4 RS 1 TL 1 ML	4 RS; 1 ML, 1 TL		Median: 20 months	Not only operated patients
Peng et al. (2022)	China	Retrospective, single-center	231	Previous surgery and or radiotherapy (*n* = 22)	6 RS; 13 ML; 217 TL; 14 TC	NA		Mean: 30 days	Not only operated patients
Perry et al. (2023)	USA	Retrospective, single-center	6	Previous surgery	3 RS; 2 TL; 1 ML	4 RS, 2 TL		Median: 59	
Roche et al. (2008)	France	Retrospective, single-center	10	Previous surgery and radiotherapy (n = NA)	4 RS; 6 TL	1 RS; 9 TL		Median 12 months	Not only operated patients
Samii et al. (2001)	Germany	Retrospective, single-center	36	Previous surgery	36 RS	36 RS		NA	No follow-up data

* Abbreviations: NA = not available; RS = retrosigmoid; ML= middle fossa; TL = translabyrinthe; TC = transcochlear

**Table 2:** Characteristics of primary and secondary outcomes

Author (Year)	GTR (*n*=)	Facial nerve deterioration (*n*=)	Hearing impairment (*n*=)	Dysphagia (*n*=)	Ischemia (*n*=)	Bleeding (*n*=)	CSF leak (*n*=)	Hydrocephalus (*n*=)	Shunt placement (*n*=)
Beatty et al. (1987)	11	7	2	1	0	1	0	NA	NA
Freeman et al. (2021)	11	15	NA	NA	1	1	3	0	0
Hong et al. (2020)	10	5	1	0	0	0	1	NA	NA
Kay-Rivest et al. (2021)	5	7	NA	NA	0	NA	2	1	NA
Kunert et al. (2014)	5	0	0	NA	0	0	1	NA	0
Nandoliya et al. (2020)	2	4	NA	NA	NA	NA	NA	NA	NA
Peng et al. (2022)	212	NA	NA	NA	NA	0	21	0	2
Perry et al. (2023)	6	1	0	0	0	0	2	NA	0
Roche et al. (2008)	6	5	NA	NA	NA	NA	NA	NA	NA
Samii et al. (2001)	36	13	0	0	NA	NA	NA	NA	NA

* Abbreviations: GTR = gross total resection; NA = not available

### Extent of resection after recurrence surgery

**Fig. 2:**
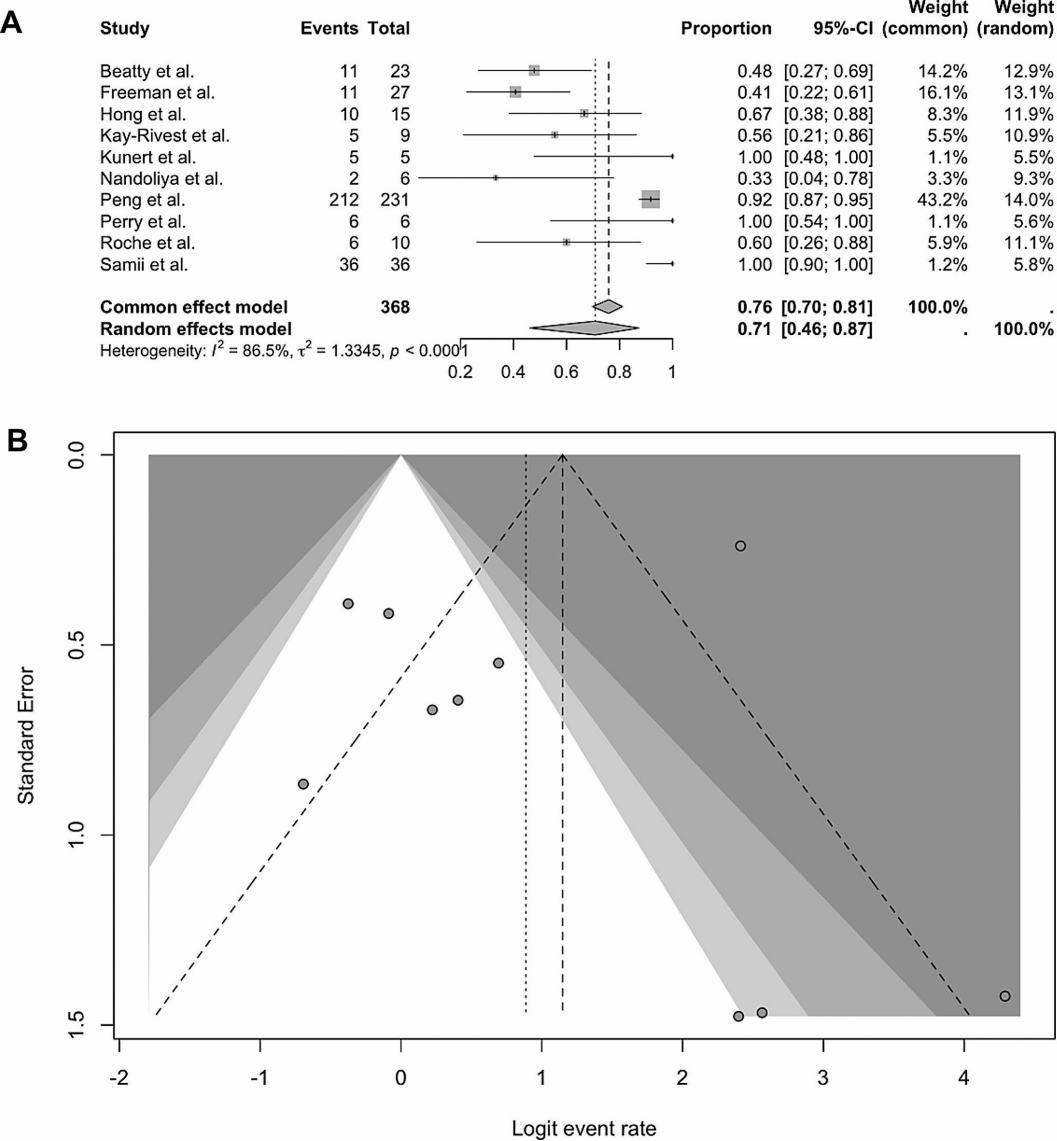
Forest Plot (**A**) and Funnel Plot (**B**) of gross total resection after microsurgery treatment for recurrent vestibular schwannoma

Across ten studies comprising **368** patients [10–12, 14–19], GTR rates ranged from **33% to 100%**. The pooled proportion of GTR was **0.71 (95% CI: 0.46–0.87)** under the random-effects model. Under the fixed-effects model, the estimate was slightly higher (**0.76; 95% CI: 0.70–0.81**), reflecting the influence of larger studies such as Peng et al. (**92%**) [15] and Freeman et al. (**41%**) [11]. Between-study heterogeneity was high (**I² = 86.5%**,** τ² = 1.3345, p < 0.0001**), indicating substantial variability in reported resection rates across studies. Figure 2 (a, b) present the corresponding forest and funnel plot.

### Progression-free survival after recurrence surgery

Four studies (49 patients) reported no tumor recurrences after revision surgery (Freeman et al. [11], Nandoliya et al. [17], Perry et al. [18], and Roche et al. [20]). Due to small samples and variable follow-up, PFS data were summarized descriptively only.

### Facial nerve function after recurrence surgery

**Fig. 3:**
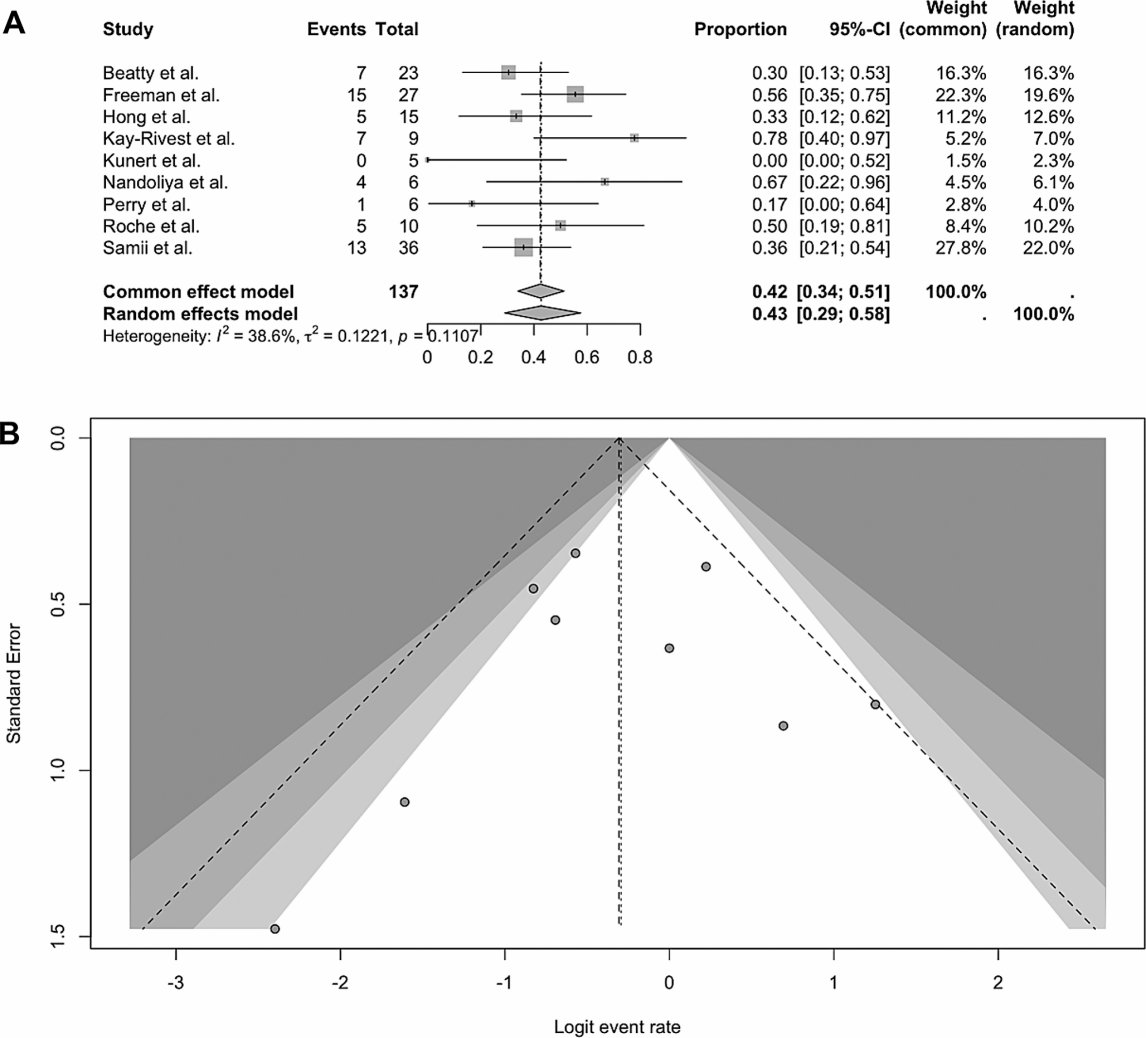
Forest plot (**A**) and funnel plot (**B**) of Facial nerve deterioration after microsurgery treatment for recurrent vestibular schwannoma

Nine studies including 137 patients reported postoperative facial nerve impairment after repeat surgery [10–12, 14, 16–19, 21]. Facial nerve deterioration was defined as postoperative deterioration of at least one point on the HB scale [7]. Assessment time points and methods varied. Beatty et al. [10] did not specify the assessment timing and used qualitative categories (normal, mild, moderate, severe, total lost) instead of the Hb scale. Freeman et al. [11] reported on the postoperative and 1-year facial outcome (used in this analysis) using the HB scale. Hong et al. [14] also uses the HB scale, but group HB 1–2, 3–4, 5–6 with no exact time point defined (while follow-up; mean 80 months). Kunert et al. [16] and Kay-Rivest et al. [21] collected data using the HB scale during follow-up (mean 33 months, respectively median 99 months). Nandoliya et al. [17] collected data using the HB in the last follow-up, with a median of 20 months. Perry et al. [18] and Roche et al. [20] measure postoperative HB without providing precise details of the timing. The study by Samii et al. [12] measures HB postoperatively and after one year, but the data do not make it entirely clear how much of the contribution comes from those who may have improved as a result of the surgery. Across these studies, facial nerve deterioration occurred in 43% of patients (pooled proportion: 0.43; 95% CI: 0.29–0.58) under a random-effects model. Individual proportions ranged from 0% to 78%, with broader confidence intervals seen in smaller cohorts. Moderate heterogeneity was present among the included studies (I² = 38.6%, τ² = 0.1221, *p* = 0.1107) with studies with larger sample sizes, such as those by Beatty et al. [10], Freeman et al. [11], and Samii et al. [12], contributed the largest weights. Figure 3 presents the corresponding forest and funnel plot.

**Fig. 4:**
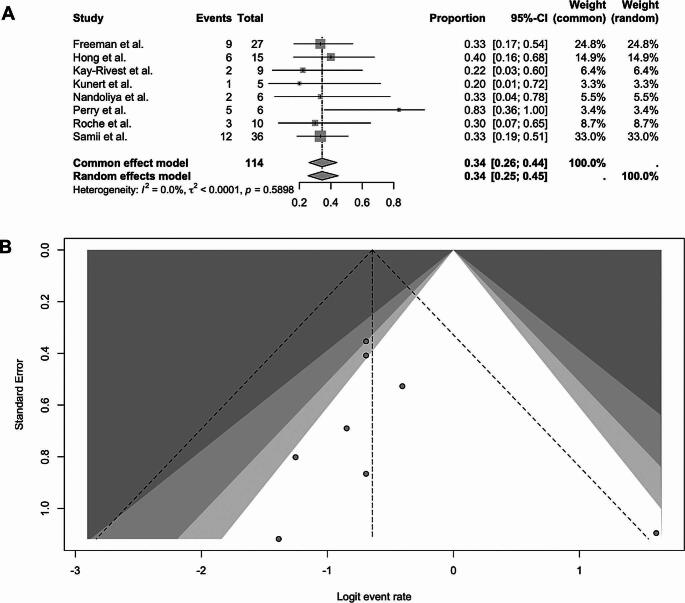
Forest plot (**A**) and funnel plot (**B**) of Facial nerve preservation (HB I-II) after microsurgery treatment for recurrent vestibular schwannoma

Analysis of favorable facial nerve outcome (House–Brackmann grade I–II) after repeat surgery included the same set of studies excluding Beatty et al. due to missing exact data regarding the HB scale. The pooled proportion of patients with good postoperative facial nerve function was 43% (random-effects model: 0.43; 95% CI: 0.29–0.58). Individual study proportions ranged from 20% to 83%, with wider confidence intervals observed in smaller cohorts. Heterogeneity across studies was negligible (I² = 0.0%, τ² < 0.0001, *p* = 0.5898). Studies with larger sample sizes, including Freeman et al. [11] and Samii et al. [12], contributed the greatest weights. The corresponding forest plot is shown in Fig. 4**.**

### Hearing impairment after recurrence surgery

Five studies including 85 patients reported postoperative hearing outcomes [10, 12, 14, 16, 18]. The pooled proportion of hearing impairment was 0.06 (95% CI: 0.03–0.14) under a random-effects model, with no heterogeneity detected (I² = 0%, τ² = 0, *p* = 0.82). Details are shown in supplementary Figure S3.

### Complications after recurrence surgery

Four studies involving 84 patients assessed postoperative dysphagia [10, 12, 14, 18]. The pooled proportion was 0.03 (95% CI: 0.01–0.09), no heterogeneity was found (I² = 0.0%, τ² = 0, *p* = 0.87). Only one event was reported, others reporting none (supplementary Figure S4).

Six studies with 146 patients reported on postoperative ischemic events [10, 11, 14, 16, 18, 21]. A single event was documented in one study (Freeman et al., 1/23; [11]), described as a cerebrovascular event, no ischemic complications were seen in others. The pooled incidence was 0.04 (95% CI: 0.03–0.07) under the random-effects model, without heterogeneity (I² = 0%, τ² = 0, *p* = 0.98, see supplementary Figure S5. Six studies (307 patients) were analysed for postoperative bleeding following revision surgery for recurrent vestibular schwannoma. Only one study [10] reported a minor postoperative hematoma not requiring treatment, while the others observed no events. The pooled incidence was 0.03 (95% CI: 0.01–0.10), without heterogeneity (I² = 0%, τ² = 0, *p* = 0.45, see supplementary Figure S6). Seven studies with 316 patients assessed postoperative cerebrospinal fluid leaks [10, 11, 14–16, 18, 21]. A total of 30 events were reported, predominantly from Peng et al. (21/231) [15], including six reoperations and two shunt placements. Additional events occurred in Freeman et al. (3/27), treated conservatively without more details [11], Hong et al. (1/15) that was treated with a lumbar drain [14] and Kunert et al. (1/5) [16], treated with a sponge dressing of the external auditory canal without need for operation or lumbar drain. Perry et al. (2/6) reported one CSF leak treated with lumbar drain and one operatively [18] and Kay-Rivest et al. [21] reported one CSF leaks without additional information of treatment. No events were recorded in Beatty et al. [10]. The pooled incidence was 0.11 (95% CI: 0.05–0.21) under the random-effects model (see supplementary Figure S7). Five studies with a total of 278 patients assessed postoperative hydrocephalus. No events were reported in Freeman et al. [11], Kunert et al. [16] or Perry et al. [18], one event was reported in Kay-Rivest et al. [21] and two in Peng et al. [15]. The pooled proportion using the random-effects model was 0.03 (95% CI: 0.00–0.22). Low heterogeneity was present (I² = 35.6%, τ² = 1.0297, *p* = 0.184, see supplementary Figure S8). Four studies comprising 269 patients reported on shunt dependency [11, 15, 16, 18]. Peng et al. documented two cases after CSF leak [15]. All other studies reported no shunt dependency. The pooled estimate was 0.02 (95% CI: 0.00–0.12) under the random-effects model with low heterogeneity observed (I² = 6.8%, τ² = 0.40, *p* = 0.36), see supplementary Figure S9 for details.

### Bias and quality evaluation

This systematic review and meta-analysis followed the NHLBI Quality Assessment Tool for Systematic Reviews and Meta-Analyses, guided by a predefined PICO question. Inclusion and exclusion criteria were defined a priori and consistently applied. A comprehensive literature search ensured broad coverage. Study selection, data extraction, and quality assessment were independently performed by two reviewers, with discrepancies resolved by consensus. Included studies were summarized narratively and in tables. Publication bias was evaluated using funnel plots for postoperative outcomes (e.g., facial nerve deterioration, hearing loss, dysphagia, ischemia, bleeding, hydrocephalus, shunt placement); no relevant asymmetry was detected, indicating low bias risk (supplementary Figure S10). Formal statistical tests were omitted due to limited study numbers. Clinical, methodological, and statistical heterogeneity were assessed using I² and Q statistics.

## Discussion

This meta-analysis shows that repeat microsurgery provides effective long-term tumor control despite technical challenges. No re-recurrences were reported, suggesting durable results comparable to primary surgery, consistent with institutional series showing > 90% recurrence-free survival after total resection [15]. However, limited follow-up, small cohorts, and publication bias overestimate control, as late recurrences can occur [2]. GTR was achieved in ~ 71%, reflecting greater technical complexity and efforts to preserve facial nerve function [22]. A comparison of surgical approaches between primary and revision procedures was limited by inconsistent and incomplete reporting across studies. While some cohorts suggested a consistent use of the same approach, others indicated a shift toward alternative approaches in the revision setting, particularly with increased use of the translabyrinthine approach. However, the lack of patient-level data precluded a systematic analysis of approach sequences. This aspect may be clinically relevant, as revision through previously operated corridors versus newly exposed anatomical pathways could influence surgical complexity and facial nerve outcomes. Facial nerve deterioration (43%) remains the essential morbidity. Low heterogeneity (I² = 38.6%) suggests this risk is intrinsic to the procedure. The proportion of patients achieving favorable facial nerve function (HB I–II) after repeat surgery indicates that good functional outcomes are attainable in a relevant subset of patients. Interpretation of these findings should, however, take into account variability in outcome assessment, particularly with regard to the timing of postoperative evaluation and differences in reporting methods across studies. In contrast, primary VS surgery achieves 85–90% preservation for smaller tumors under more favorable conditions [22, 23]. Re-operations are complicated by scar tissue, distorted anatomy, and loss of arachnoid planes, making nerve identification and preservation difficult [11, 12].

Intraoperative neuromonitoring improves outcomes in primary VS surgery [24], but was rarely reported here—only Hong et al. [14] and Kunert et al. [16] used continuous monitoring. Surgical approach influences outcomes [Ansari, 2012 #2603]; most studies used the retrosigmoid route, data on patient positioning and tumor size were insuffizient for subgroup analysis. Planned near-total resection may balance tumor control and facial nerve preservation in complex cases [25]. Postoperative hearing deterioration was rare. However, this likely reflects selection bias, as preoperative hearing was often absent. Hearing preservation in settings where patients are pre-treated remains uncommon, with primary VS series reporting hearing preservation rates often below 25%, especially for larger tumors or poor preoperative hearing [26–28].

Repeat microsurgery demonstrated an acceptable safety profile. Ischemia (4%), hemorrhage (3%), dysphagia (3%) and hematoma (3%) were infrequent, supporting the safety of salvage surgery following primary treatment in experienced skull base centers. However, definitions and diagnostic methods varied across studies, limiting comparability and possibly underestimating subclinical events. Cerebrospinal fluid (CSF) leakage represented the most frequent adverse event, affecting about 11% of patients—comparable to rates in large primary series (~ 9–12%) [29, 30]. CSF leaks are a well-known risk in VS surgery, particularly in revision cases where watertight dural closure is challenging. The predominance of leaks in the large cohort of Peng et al. [15] likely reflects more detailed reporting rather than a true increase in risk. Low heterogeneity across studies suggests consistent incidence rates. Advances in closure techniques and the use of postoperative lumbar drainage can further reduce this risk [30, 31]. Hydrocephalus was observed in 3% of patients, while shunt dependency occurred in approximately 2% of patients, typically secondary to persistent CSF leakage. Although rare, such complications carry notable morbidity and require vigilance for delayed CSF flow disturbances. Incomplete reporting across studies may underestimate the incidence.

Stereotactic radiosurgery (SRS) remains valuable for smaller or stable recurrences, achieving ~ 95% control at 5 years, though with delayed neuropathy risk [4, 32]. By contrast, microsurgery allows definitive tumor removal, decompression and histological confirmation, maintaining its role for large, symptomatic, or post-radiation recurrences.

A multidisciplinary approach balancing oncologic control and facial nerve function is recommended. Future research should focus on multicenter registries employing standardized definitions (especially “recurrence” vs. “residual”), uniform House–Brackmann reporting, and consistent long-term follow-up. Such harmonization will be essential for improving evidence quality and refining treatment strategies in this challenging patient population.

### Limitations

Interpretation is limited by heterogeneous and incomplete datasets. Possible inclusion of NF2-related schwannomatosis cases cannot be entirely excluded. Although the NOS assessment indicated variable methodological quality among studies, all eligible data were retained to preserve statistical power and representativeness, given the rarity of recurrent vestibular schwannoma. Additionally, definitions of “residual” versus “recurrent” tumor varied across studies, introducing potential misclassification bias. Because all included studies were retrospective case series without control groups, the analysis was restricted to prevalence-based estimates rather than comparative measures such as odds ratios. A notable limitation is the dominance of single large studies (e.g., Peng et al. [15]) in specific outcomes such as CSF leakage, which disproportionately influenced the pooled complication rates. Furthermore, the absence of longitudinal follow-up data precluded evaluation of long-term functional recovery or delayed tumor regrowth beyond the reported observation periods. Despite these constraints, this analysis provides the most comprehensive overview to date of microsurgery for recurrent pre-treated VS after.

## Conclusion

This meta-analysis demonstrates that repeat microsurgical resection can achieve high GTR rates and durable tumor control, albeit with a non-negligible risk of postoperative morbidity, particularly regarding facial nerve function. Facial nerve deterioration remains the key functional risk, while CSF leakage and shunt dependency are uncommon.

When performed in specialized skull base centers with modern monitoring, microsurgery offers safe and lasting outcomes for selected patients with recurrent vestibular schwannoma.

## Supplementary Information

Below is the link to the electronic supplementary material.

Supplementary Material 1

Supplementary Material 2

## Data Availability

The data sets generated and analyzed in the current study are available upon request from the corresponding author.
