# Supplementary material for: Salvage surgery following primary treatment in recurrent vestibular schwannoma: surgical outcomes and progression-free survival- a meta-analysis
Source: J Neurooncol. 2026 May 29;178(1):34. doi: 10.1007/s11060-026-05629-w (PMC13221319; doi:10.1007/s11060-026-05629-w)
Supplement: Supplementary file 1 — Supplementary Material 1 [file 11060_2026_5629_MOESM1_ESM.docx]

**Supplementary**

Surgical Outcomes and Progression-Free Survival in Recurrent Vestibular Schwannoma: A Meta-Analysis

Lisa Haddad ^1,2*^, Felix Arlt^1,2^, Erdem Güresir ^1,2^, Johannes Wach ^1,2^

^1^Department of Neurosurgery, University of Leipzig Medical Center, 04103 Leipzig, Germany

^2^Comprehensive Cancer Center Central Germany, Partner Site Leipzig, 04103, Leipzig, Germany.

Corresponding Author:

Lisa Haddad

Leipzig University, University Hospital Leipzig, Department of Neurosurgery Liebigstraße 20, 04103 Leipzig, Germany

Lisa.haddad@medizin.uniklinik-leipzig.de

Tel.: +49-341-97 17500

ORCID:

Lisa Haddad - <https://orcid.org/0009-0004-3135-495X>

Felix Arlt - <https://orcid.org/0000-0003-3749-2494>
Erdem Güresir - <https://orcid.org/0000-0001-8319-0847>

Johannes Wach - <https://orcid.org/0000-0002-4680-0412>


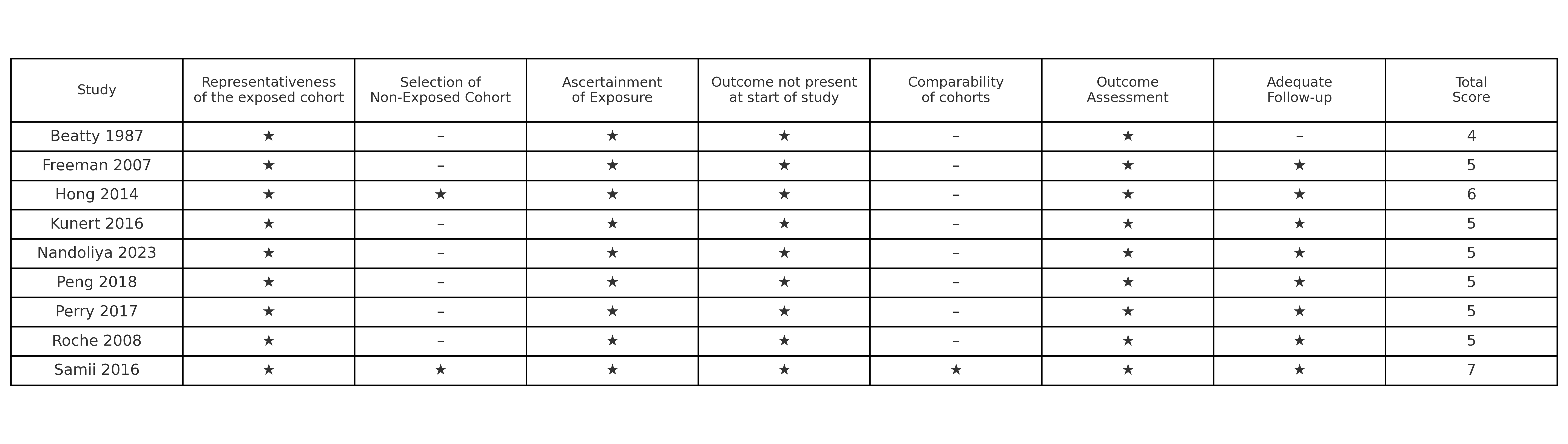


**Supplementary Table S2:** Risk of bias using the Newcastle-Ottawa Scale (NOS) for non-randomized studies.

**
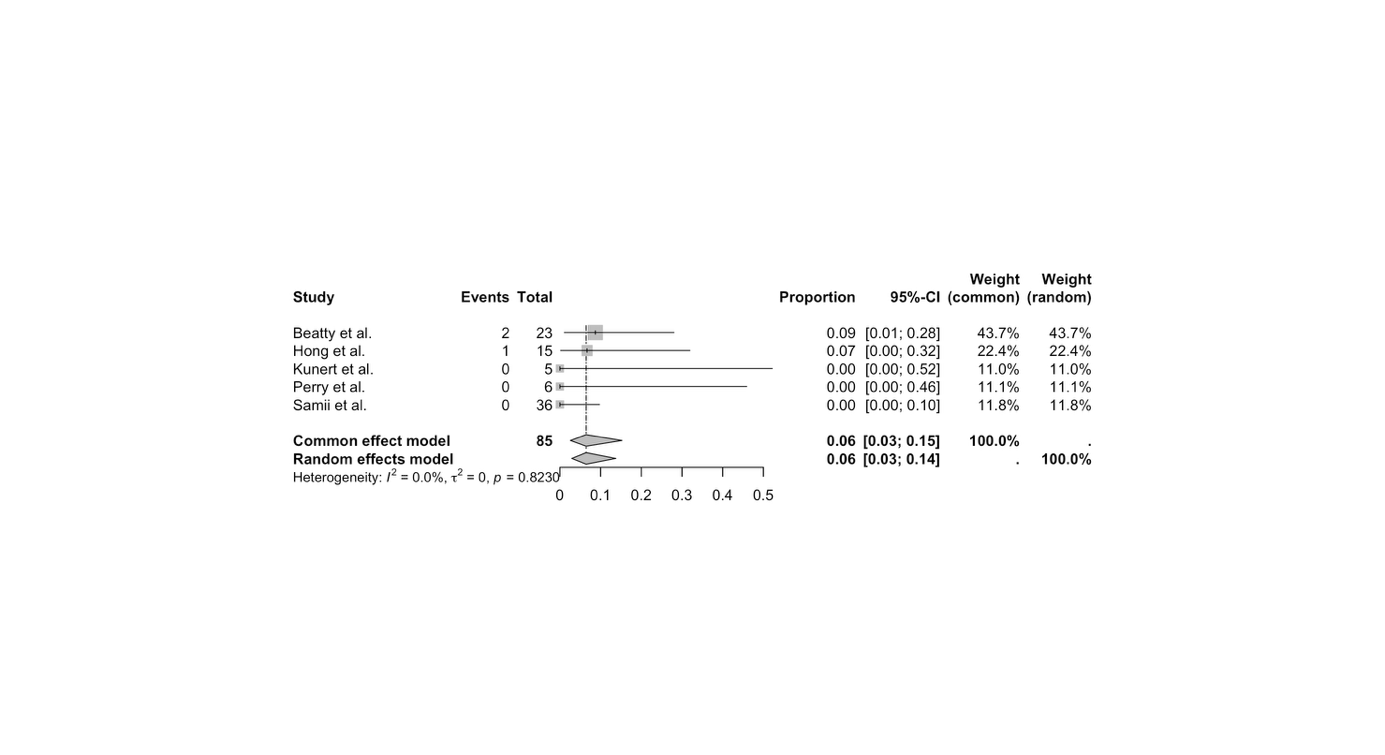
**

**Fig. S3.** Forest Plot of Hearing impairment after re-do surgery for recurrent vestibular schwannoma.

**
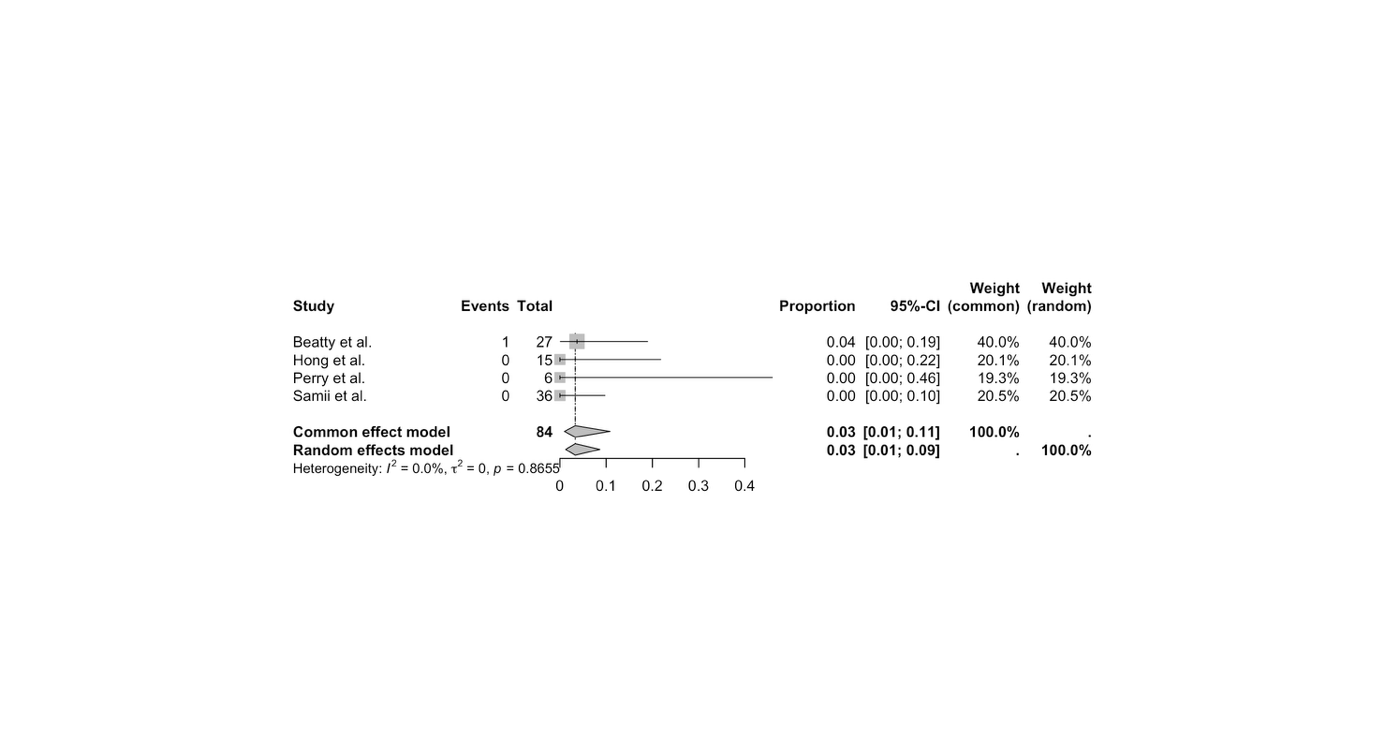
**

**Fig. S4.** Forest Plot of dysphagia after re-do surgery for recurrent vestibular schwannoma.


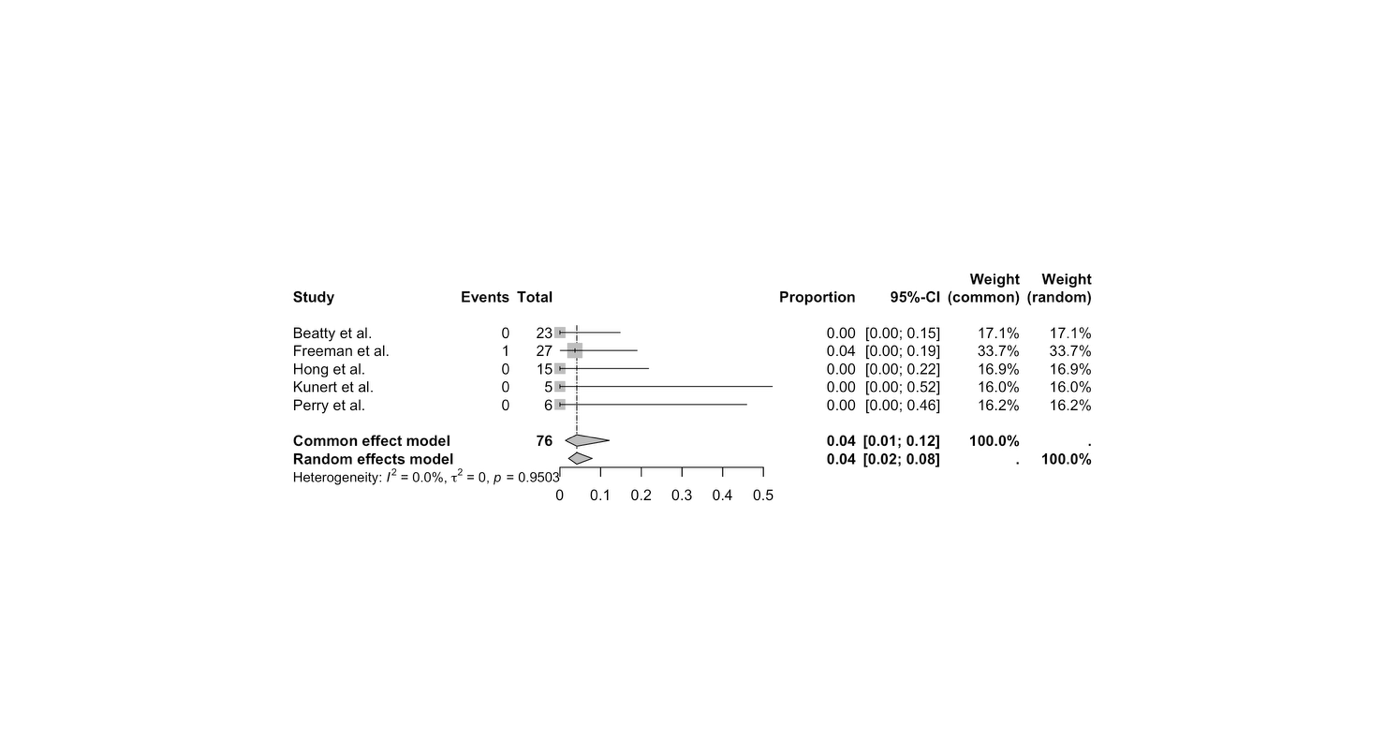


**Fig. S5.** Forest Plot of ischemia after re-do surgery for recurrent vestibular schwannoma.





**Fig. S6.** Forest Plot of bleeding after re-do surgery for recurrent vestibular schwannoma.


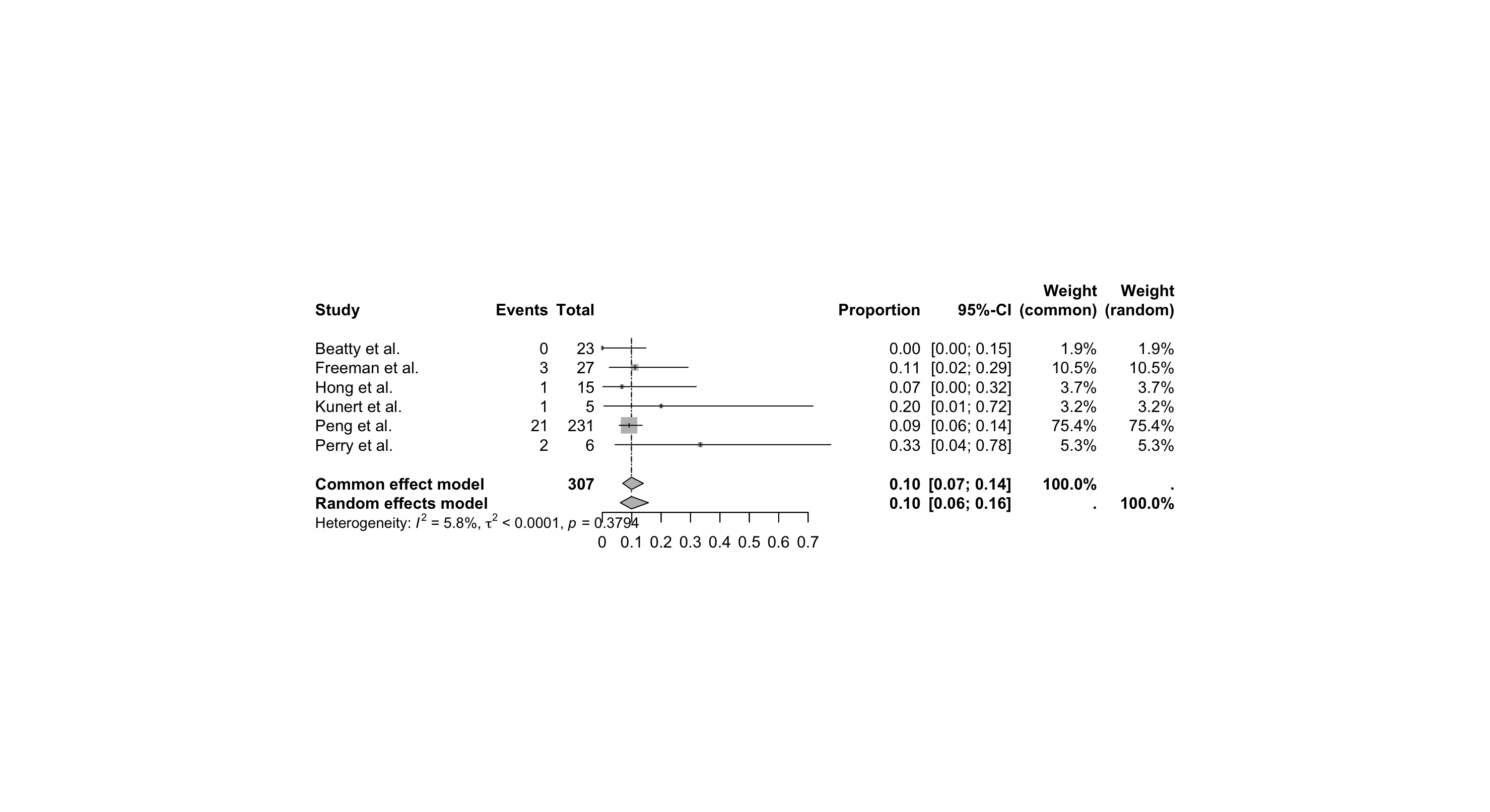


**Fig. S7.** Forest Plot of CSF leak after re-do surgery for recurrent vestibular schwannoma.


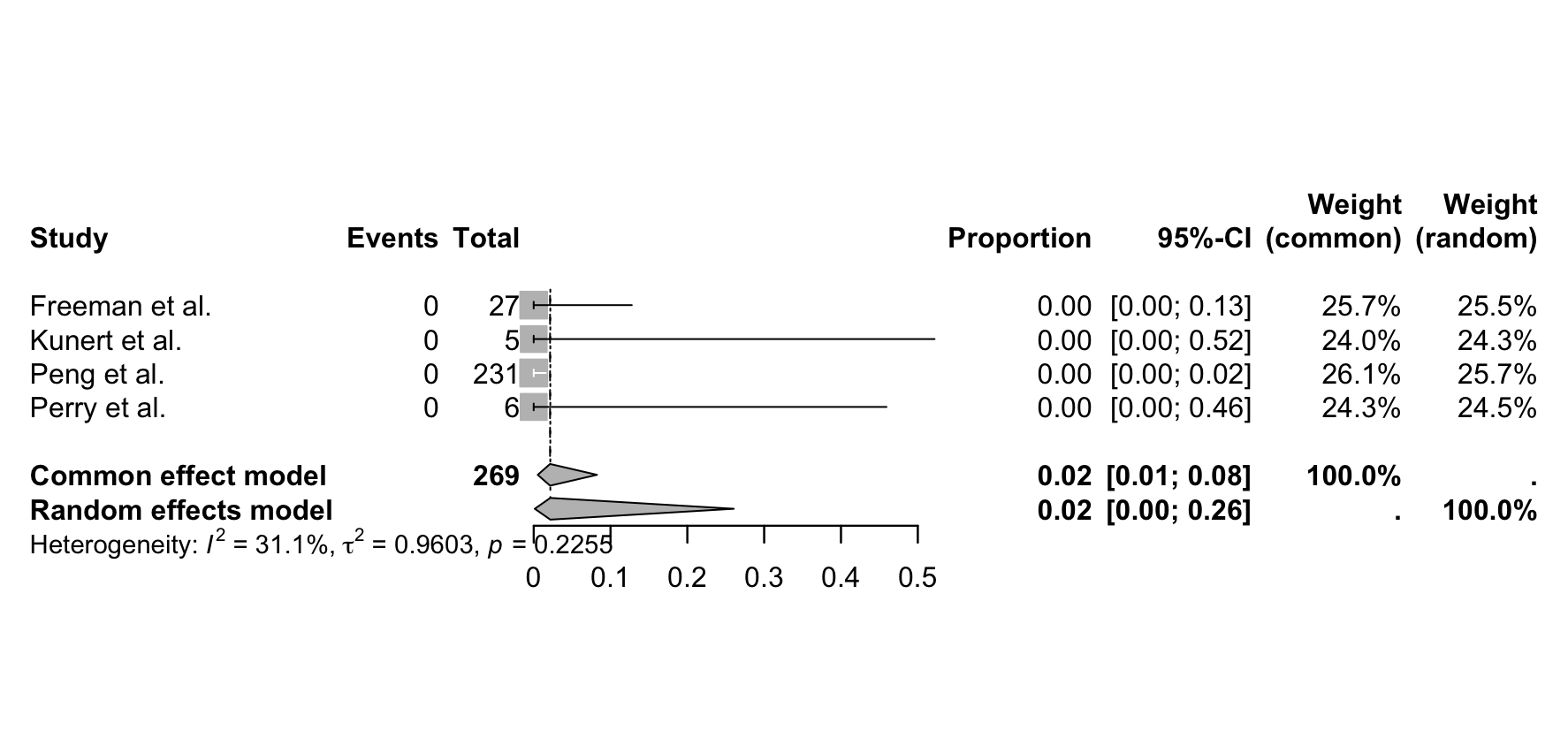


**Fig. S8.** Forest Plot of hydrocephalus after re-do surgery for recurrent vestibular schwannoma.


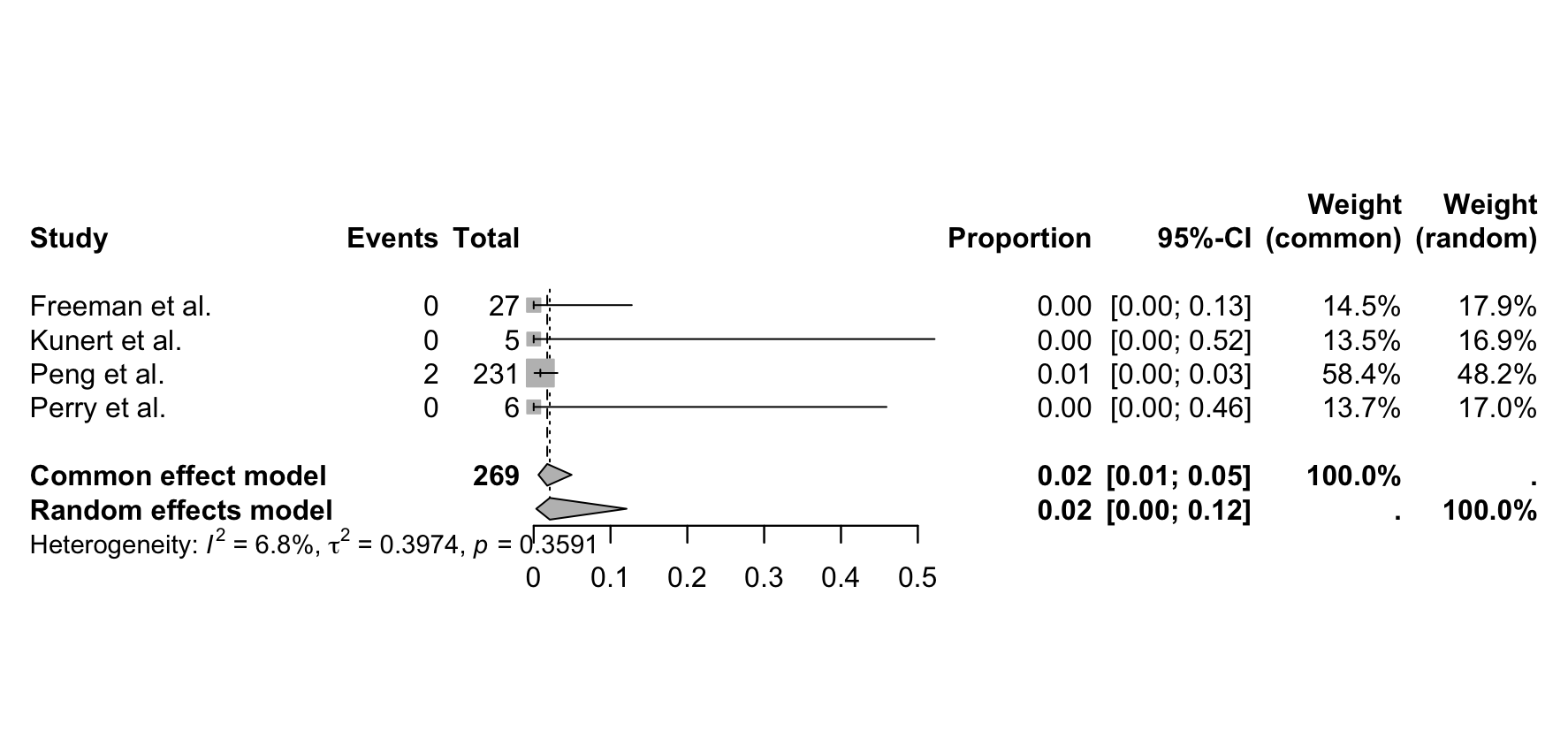


**Fig. 9.** Forest Plot of shunt dependency after re-do surgery for recurrent vestibular schwannoma.

**
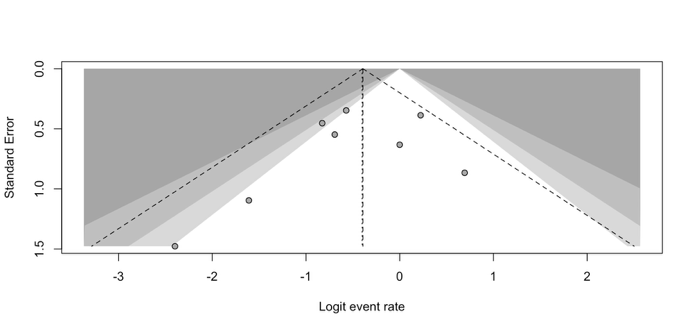

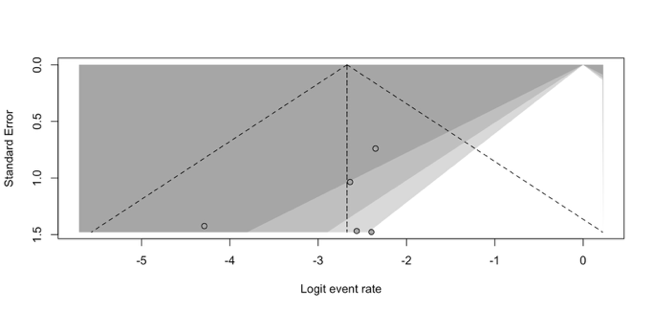
A B**

**
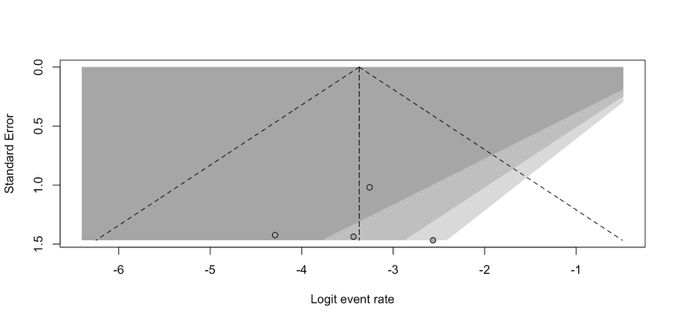

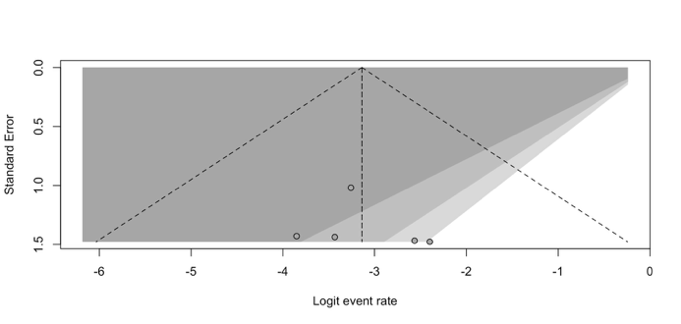
**

**C D**

**
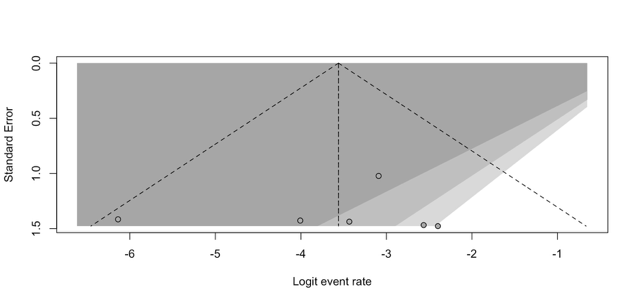

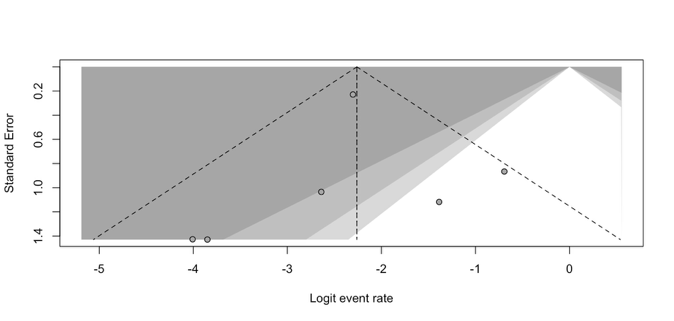
**

**E F**

**
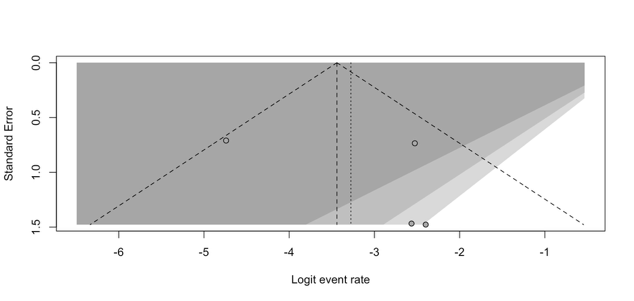

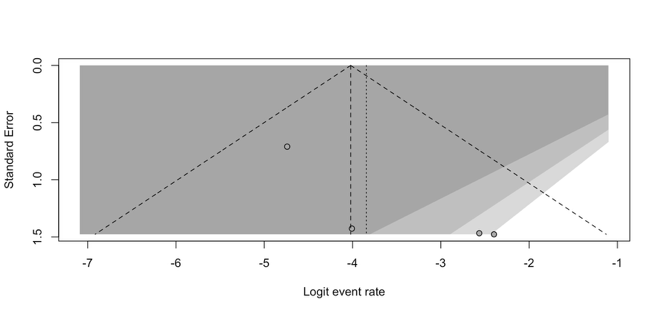
**

**G H**

**Fig. S10:** Funnel plots for the secondary endpoints of the present meta-analysis: Facial nerve deterioration **(A)**; hearing impairment **(B)**; dysphagia **(C)**; ischemia **(D)**; bleeding **(E)**; CSF leak **(F)**; hydrocephalus **(G)**; Shunt placement **(H).**
